# Anti-TNF-Related Medium-Vessel Vasculitis: A Report of a Rare Adverse Drug Reaction

**DOI:** 10.7759/cureus.93342

**Published:** 2025-09-27

**Authors:** Mehrnoush Hassas Yeganeh, Arooba Malik, Aya Yaseen, Hamidreza Zefreh, Daniel Shostak, Sajina Prabhakaran

**Affiliations:** 1 Internal Medicine, Capital Health Regional Medical Center, Trenton, USA; 2 School of Medicine, Isfahan University of Medical Sciences, Isfahan, IRN

**Keywords:** autoimmunity, case report, certulizumab, inflammation, vasculitis

## Abstract

Tumor necrosis factor (TNF) inhibitors have significantly improved outcomes for patients with rheumatoid arthritis (RA). However, these biologic agents can lead to rare but serious adverse events, including drug-induced vasculitis. We report a rare case of anti-TNF-induced medium-vessel vasculitis in a 33-year-old female with seronegative RA and a complex medical history. After inadequate responses and adverse effects from previous treatment, the patient was switched to anti-TNF certolizumab pegol. Shortly after initiation, she developed painful, erythematous, and pruritic rashes on her hands and arms. A skin biopsy confirmed medium-vessel vasculitis. The condition improved following the discontinuation of certolizumab and initiation of immunosuppressive therapy with corticosteroids. This case underscores the importance of vigilance for dermatologic adverse effects in patients receiving TNF inhibitors. Vasculitis should be considered as a differential diagnosis when new skin lesions appear during biologic therapy, and management should be initiated accordingly.

## Introduction

Rheumatoid arthritis (RA) is a known chronic autoimmune condition that can be managed through different ways, including biologic therapies like tumor necrosis factor (TNF) inhibitors, which have significantly improved clinical outcomes [1-3]. However, these biologic agents are not without adverse effects. Certolizumab pegol (such as Cimzia), a commonly used TNF inhibitor, has been associated with rare dermatologic reactions, including drug-induced vasculitis, which are mostly categorized as small-vessel vasculitis [4,5].

Although nearly 16 cases reported to develop medium-vessel vasculitis after different anti-TNF treatments (adalimumab, infliximab, etanercept) [6], due to lack of specific and detailed reporting of each cases, its true incidence rate, separate relationships between biologic agent, auto-immune disease, and dermatologic finding, and exact index date for initiation of skin probblems are not fully understood [6].

This case report presents medium-vessel vasculitis with certulizumab pegol for the first time in a patient with seronegative RA, emphasizing that both patients and healthcare providers should be aware of this complication and talk about it with their patients. This case highlights the importance of early recognition and effective management of rare adverse effects in patients undergoing biologic therapy.

## Case presentation

The patient is a 33-year-old female with a complex medical history, including seronegative RA, obesity, depression, gastroesophageal reflux disease, infertility, hypermobile joints, and lumbar radiculopathy. She was diagnosed with RA (seronegative, positive ANA with 1:320 titration and speckled pattern, no hypocomplementemia, negative anti-phospholipid syndrome antibodies, no TB, negative for hepatitis B virus (HBV)/hepatitis C virus (HCV)/human immunodeficiency virus (HIV), normal kidney/liver/thyroid functions) in her late twenties, which presented with typical inflammatory symptoms. Initially, her condition was managed with sulfasalazine, but the medication was discontinued due to nausea and the development of a malar-like rash. She was then switched to methotrexate, which was poorly tolerated due to mucositis, leading to the introduction of biologic therapy. Her chronological order of changes in medications and clinical notes after her visits with the physician is mentioned in Table 1.

**Table 1:** Chronological flow of events in the patient

Date of visit (MM/YY)	Notes
5/22	-Adalimumab started. -Tolerated well through 2022
3/23	-Overall pain in the hand (severity: 8-9/10) with tingling and numbness, tenderness over the metacarpophalangeal joints, morning stiffness for 10-15 min, better with movement
4/23	-Hands feel better (severity: 5/10), prone to dropping things, feel getting stuck (trigger finger), positive stiffness
7/23	-Left hand pain (severity: 4/10) with numbness and tingling
11/23	-Pain throughout the body, 4^th^ digit is the worst, cold weather makes it worse, lower back pain and stiffness, cannot bend forward, jaw pain at times, -Discussing new medication
1/24	-Pain and tenderness over metacarpophalangeal joints of the left hand, jaw pain and point tenderness, still having back pain and stiffness, -Adalimumab stopped, and Etanercept started
5/24	-Etanercept is well-tolerated, feeling good, no pain, 5 min morning stiffness
6/24	-Worsening left knee pain started 4 days ago, -Pain and swelling of the left knee, ankle, wrist, difficulty climbing stairs, pain level 8/10, -Prednisone for 3 days+Knee sleeve, -Continue Etanercept
6/24	-Persistent left knee pain with slight warmth overlying the patellar tendon, radiating to the foot and hip, discomfort on the lateral aspect as well as the supra-patellar fossa without effusion or synovitis, discomfort with flexion and extension, and rolling over, -Triamcinolone intra-articular injection+continue Etanercept
8/24	-Knee pain is better after a corticosteroid -Joint pains recur after 3-4 days of Etanercept injection, -Thinking of switching to another biologic agent, -Continue Etanercept till further notice by the insurance
1/25	Etanercept was stopped, and Certolizumab was started due to inadequate and unsatisfactory response to Etanercept
2/25	-Good tolerance to injection, -Starting some skin rash, -Suggest anti-histamine+topical medications (assumed it is an injection reaction or a simple dermatologic allergic reaction), -Dermatology visit if it continues, -Certolizumab was stopped
3/25	-Persistent, raised, painful, erythematous rash, - Punch biopsy was taken and reported as medium-vessel vasculitis, -Responded to systemic corticosteroid

The patient began adalimumab, which initially provided relief from her RA symptoms. However, over time, her joint pain worsened, and she required an increase in the frequency of her injections. Despite this adjustment, she continued to experience disease progression and developed tender and swollen joints(relief lasting less than one week). After several months of unsatisfactory response to adalimumab, she switched to etanercept. Again, this regimen was initially working for her, but after a time, joint pain recurred; she continued to experience flares, and she was feeling well for not more than a couple of days after the etanercept injection (Table 1).

**Figure 1:**
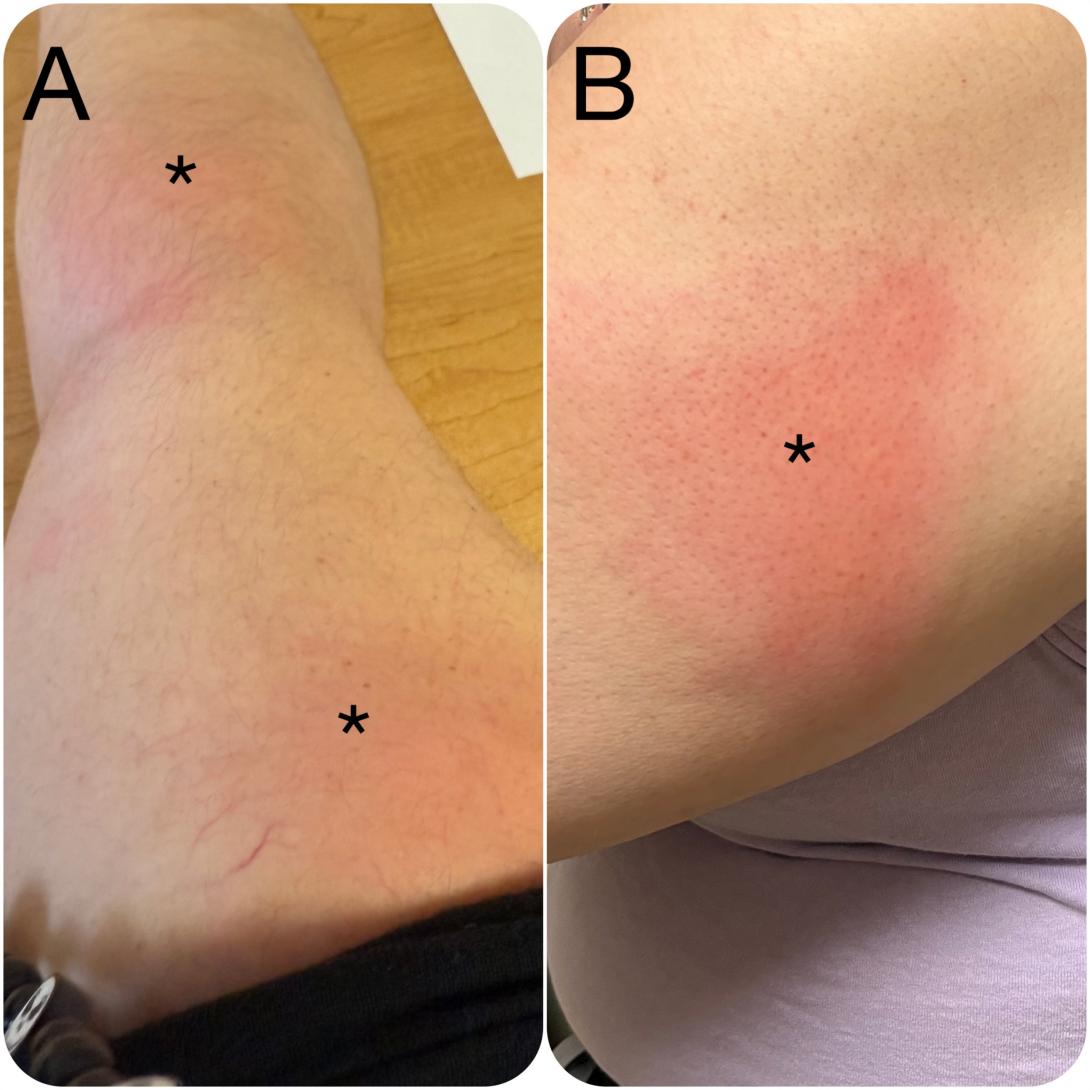
Anti-TNF-induced erythema in the upper limb The affected parts are shown by the asterisks (*). TNF: tumor necrosis factor

To better control her symptoms and due to unsatisfactory results with adalimumab and etanercept, after consultation and considering all of the aspects, she transitioned to certolizumab pegol. Initially, the treatment seemed promising, but soon after, she began experiencing new skin symptoms. These included the development of erythematous, raised, and painful rashes on her hands and arms (Figure 1). The rashes were accompanied by swelling and burning, particularly affecting the fingers. Over time, these lesions became persistent, and she reported discomfort and pruritus in the affected areas. A punch skin biopsy and dermatophathologic evaluations revealed superficial and deep perivascular and interstitial brisk lymphocyte infiltration, evidence of eosinophil infiltration, and medium-vessel vasculitis with fibrinoid necrosis (ANCA was negative), which raised concerns that certolizumab may be the causative agent. After its cessation and initiation of corticosteroids intravenously, the symptoms subsided successfully.

## Discussion

Biologic agents, such as TNF inhibitors, are a mainstay in the treatment of RA and other inflammatory conditions, such as inflammatory bowel disease or psoriasis, offering significant therapeutic benefits [1,4]. However, their use has been linked to a variety of adverse effects, including dermatologic complications [5]. Certolizumab pegol, a TNF inhibitor, is generally well-tolerated but has been rarely associated with skin reactions, mostly in the category of leukocytoclastic vasculitis [5-8]. This case represents one such event of certolizumab-induced medium-vessel vasculitis, contributing valuable insights to the dermatologic side effects of biologic therapies.

Dermatologic reactions to anti-immune medications

Biologic medications, particularly those targeting TNF-α, play a crucial role in suppressing the immune system, reducing inflammation, and preventing joint damage in autoimmune diseases such as RA [7,8]. However, these therapies can also induce immune dysregulation, leading to a spectrum of skin reactions, ranging from mild rashes to severe vasculitis. Dermatologic reactions to TNF inhibitors typically include erythematous rashes, urticaria, and, in rare cases, vasculitis [5,8-10].

Vasculitides associated with biologic agents can be categorized based on the size of the affected blood vessels. Most of the previously reported anti-TNF-related dermatologic problems were small-vessel vasculitides [5]. Medium-vessel vasculitis typically involves vessels with diameters between 100 µm and 1 mm. It is characterized by inflammation of the medium-sized arteries, leading to a wide range of skin manifestations such as polymorphous rash, purpura, livedo reticularis, ulcers, and erythematous nodules [5,6]. In this case, certolizumab pegol likely triggered an immune-mediated response, manifesting as medium-vessel vasculitis, evidenced by the patient's erythematous, raised rashes and painful swelling, especially in the hands and arms (Figure 1); something that has not been found as a complication of certolizumab [5,6].

Mechanisms and etiology of biologic-induced skin reactions

The mechanisms behind TNF inhibitor-induced dermatologic reactions, particularly vasculitis, are not fully understood, but several hypotheses have been proposed:

Immune System Dysregulation

TNF-α regulates immune responses, particularly inflammation and the activation of immune cells. By inhibiting TNF-α, biologic therapies like certolizumab can disrupt this balance, leading to autoimmune-like responses [4,11]. These disruptions can lead to the development of autoimmunity, such as drug-induced lupus erythematosus (DILE) or vasculitis, including medium-vessel involvement. The inhibition of TNF-α may promote antibody production against self-antigens, leading to inflammation in blood vessels and other tissues [11,12].

Immune Complex Formation

The binding of TNF inhibitors to TNF-α can form immune complexes that deposit in the walls of blood vessels. These complexes trigger local inflammatory reactions (hypersensitivity type 3), leading to vasculitis. The subsequent activation of complement pathways and immune cells can result in vessel wall damage and the clinical manifestation of vasculitis, as seen in this case [9].

Direct Endothelial Damage

TNF inhibitors, through their effects on the immune system, may also lead to direct endothelial cell injury. This endothelial damage can compromise the integrity of the vessel walls, leading to inflammation and vascular permeability, thrombosis, and potentially causing skin lesions and other systemic manifestations [13,14].

Molecular Mimicry

Another potential mechanism is molecular mimicry, in which the immune system mistakenly targets healthy tissues following an infection or exposure to a drug. This can lead to autoimmune vasculitis triggered by biologic agents, for example, certolizumab in our case. Molecular mimicry has been implicated in the pathogenesis of drug-induced vasculitis; however, further research is needed to understand its role in TNF inhibitor-induced reactions fully [15].

Vasculitis and its clinical presentation

The presentation of medium-vessel vasculitis in the context of TNF inhibitors often includes different patterns of erythematous rash, skin ulcers, or nodules [5,6]. The differential diagnosis of these dermatological manifestations could be an allergic reaction to the injection, rheumatoid skin disease, or primary systemic medium-vessel vasculitis. Most reported cases of anti-TNF-induced vasculitis were in the small-vessel category [5]. Anti-TNF-induced medium vessel vasculitis has been reported in three studies before this: two case series, each with eight and seven patients, and one case report [6]; the biologic agent was not certolizumab in any of them. They reported that the mean interval between the initiation of treatment and vasculitis presentation was 9 months (range, 0.5-80 mo). For final diagnosis and treatment, a biopsy confirms the underlying offending pathology. In our case, one month after injection, she presented with painful rashes on her hands and arms, which were raised, erythematous, and accompanied by burning and swelling. These rashes, along with the punch biopsy showing superficial and deep lymphocytic and eosinophilic infiltration and medium-vessel vasculitis, were consistent with an allergic, immune-mediated reaction to Certolizumab pegol. The absence of systemic involvement, such as pulmonary or renal manifestations, points to a localized, skin-limited form of vasculitis.

Other medications associated with medium-vessel vasculitis

Biologic Agents

Similar to TNF inhibitors, other biologic agents, such as rituximab and tocilizumab (IL-6 inhibitors), have been reported to induce vasculitis in some patients. The mechanism likely involves immune dysregulation and the formation of immune complexes that target vascular tissues.

Antibiotics

Certain antibiotics, particularly beta-lactams, sulfonamides, and fluoroquinolones, have been associated with an increased risk of vasculitis. These drugs can trigger hypersensitivity reactions, leading to immune complex deposition in medium-sized blood vessels [16].

Hydralazine and Minocycline

Both hydralazine (an antihypertensive agent) and minocycline (an antibiotic used in dermatological conditions) have been implicated in drug-induced lupus and vasculitis. These drugs can cause autoimmune reactions, particularly in genetically predisposed individuals [16].

Checkpoint Inhibitors

Immune checkpoint inhibitors, such as nivolumab and pembrolizumab, used in cancer immunotherapy, have been associated with vasculitis due to their ability to activate immune responses. These agents have been reported to trigger immune-mediated vasculitis, including medium-vessel vasculitis, particularly in patients with underlying autoimmune conditions [17].

Management of drug-induced vasculitis

The management of drug-induced medium-vessel vasculitis primarily involves discontinuing the potentially problematic medication and giving sufficient time to the body to wash out the offending agent [6,18-20]. Treatment often includes the use of immunosuppressive agents, such as prednisone, which was utilized in this case to control inflammation. IV corticosteroids are commonly used in severe cases to provide rapid symptom relief [6,18-20]. In addition, symptomatic treatments, such as antihistamines, may be used to address pruritus, although these may not fully address the underlying vasculitis. Close monitoring is essential to ensure that skin lesions and other inflammatory manifestations resolve and that no systemic involvement develops [6,18-20].

## Conclusions

This case report demonstrates a rare but significant complication of certolizumab therapy: drug-induced medium-vessel vasculitis. While TNF inhibitors are effective in treating rheumatoid arthritis, they are associated with a range of adverse effects, including dermatologic reactions such as vasculitis. Although not fully understood, clinicians and patients who are taking these agents should be alert for signs of drug-induced vasculitis and other dermatologic manifestations, particularly when receiving long-term biologic treatments.
